# Targeting Cognition and Behavior Post-Stroke: Combined Emotional Music Stimulation and Virtual Attention Training in a Quasi-Randomized Study

**DOI:** 10.3390/brainsci15111168

**Published:** 2025-10-29

**Authors:** Rosaria De Luca, Federica Impellizzeri, Francesco Corallo, Andrea Calderone, Rosalia Calapai, Alessio Mirabile, Lilla Bonanno, Maria Grazia Maggio, Angelo Quartarone, Irene Ciancarelli, Rocco Salvatore Calabrò

**Affiliations:** 1 IRCCS Centro Neurolesi Bonino Pulejo, 98124 Messina, Italy; rosaria.deluca@irccsme.it (R.D.L.); francesco.corallo@irccsme.it (F.C.); andrea.calderone@irccsme.it (A.C.); rosalia.calapai@irccsme.it (R.C.); alessio.mirabile@irccsme.it (A.M.); lilla.bonanno@irccsme.it (L.B.); mariagrazia.maggio@irccsme.it (M.G.M.); angelo.quartarone@irccsme.it (A.Q.); roccos.calabro@irccsme.it (R.S.C.); 2 Department of Life, Health and Environmental Sciences, University of L’Aquila, 67010 L’Aquila, Italy; irene.ciancarelli@univaq.it; 3 Territorial Rehabilitation Department, ASL Avezzano-Sulmona-L’Aquila, 67100 L’Aquila, Italy

**Keywords:** neurorehabilitation, chronic stroke, music stimulation, virtual reality training, attention processes, intrinsic motivation, mood symptoms, autonomic regulation

## Abstract

**Background**: Emotionally salient music may enhance attention-focused rehabilitation, yet concurrent music plus virtual-reality programs in chronic stroke are largely untested. We assessed whether personalized emotional music stimulation (EMS) layered onto a standardized virtual reality rehabilitation system (VRRS) augments cognitive, affective, physiological, and functional outcomes. **Methods**: In a quasi-randomized outpatient trial, 20 adults ≥ 6 months post-ischemic stroke were allocated by order of recruitment to VRRS alone (control, *n* = 10) or VRRS+EMS (experimental, *n* = 10). Both groups performed 45 min of active VRRS cognitive training (3×/week, 8 weeks), while the EMS group received approximately 60 min sessions including setup and feedback phases. Primary outcomes were cognition and global function; secondary outcomes were intrinsic motivation, depression, anxiety, and heart rate. Non-parametric tests with effect sizes and Δ-scores were used. **Results**: The experimental group improved across all domains: cognition (median +4.5 points), motivation (median +54 points), depression (median −3.5 points), anxiety (median −4.0 points), heart rate (median −6.35 beats per minute), and disability (median one-grade improvement), each with large effects. The control group showed smaller gains in cognition and motivation and a modest heart-rate reduction, without significant changes in mood or disability. At post-treatment, the music group outperformed controls on cognition, motivation, and disability. Change-score analyses favored the music group for every endpoint. Larger heart-rate reductions correlated with greater improvements in depression (ρ = 0.73, *p* < 0.001) and anxiety (ρ = 0.58, *p* = 0.007). **Conclusions**: Adding personalized emotional music to virtual-reality attention training produced coherent, clinically relevant gains in cognition, mood, motivation, autonomic regulation, and independence compared with virtual reality alone.

## 1. Introduction

Stroke, with its main ischemic and hemorrhagic subtypes, continues to represent a major global health burden, ranking among the leading causes of adult morbidity and mortality [1,2,3]. The burden of stroke worldwide is growing, producing more than 12 million new cases each year, and the lifetime risk has shifted dramatically higher, with one in every four adults at risk developing the condition [4]. Acute treatments for stroke have improved, leading to decreased early mortality; however, the prevalence of chronic poststroke sequelae is on the rise and poses a profound burden on individuals, families, and society, as well as on the global economy [5,6].

Recovery after stroke unfolds across several stages: the acute phase (hours to days), focused on immediate stabilization; the subacute phase (days to weeks), targeting early rehabilitation and complication prevention; the post-acute phase (weeks to months), characterized by intensive neurorehabilitation; and the chronic phase (generally beyond six months), where persistent deficits become the target of long-term adaptation strategies [7,8,9]. It is in the chronic phase that the limits of spontaneous neurological recovery become most apparent, underscoring the need for sustainable, patient-centered interventions [10]. Cognitive and behavioral sequelae depend on the location and extent of the damage [11], ranging in severity from mild to severe, and occur in up to 60% of stroke survivors in the first year after stroke [12]. Notable syndromic profiles include post-stroke depression (PSD), anxiety, apathy, and poor compliance to treatment, all of which can substantially affect rehabilitation success [13,14]. The fronto-parietal cortex has been consistently implicated in attentional control processes [15]. Pronounced dysregulation of the prefrontal cortex (PFC) and anterior cingulate cortex (ACC) is characteristic of multiple anxiety and mood disorders, and the restoration of their function is a critical indicator of therapeutic efficacy [16]. In the primate brain, attentional selection in the visual domain is mediated by a large-scale network of regions within the thalamus and occipital, temporal, parietal, and frontal cortex [17]. Lesions of the right fronto-parietal region are significantly correlated with deficits in visuo-spatial attention, praxis, spatial awareness, and tactile perception [15,16,17,18]. These neuropsychological and emotional disturbances are not merely secondary consequences or reactions to injury; rather, they constitute the brain’s primary interpretation and outward expression of the insult, with profound implications for motivation, self-agency, and quality of life [19].

The assessment of stroke survivors is multidimensional, involving clinical scales (i.e., modified Rankin Scale (mRS)) [20], behavioral scales (such as the Hamilton Rating Scale for Depression (HRS-D) [21] and Anxiety (HRS-A) [22]), and cognitive tools (such as the Montreal Cognitive Assessment (MoCA)) [23]. Pharmacological treatment, as in chronic stroke, remains a multimodal approach primarily targeting mood disorders, complemented by a growing list of non-pharmacological interventions [24]. The delivery method of cognitive rehabilitation, which in the past was performed with pen and paper or through face-to-face interviews, is now changing thanks to technological advances (e.g., software, tools, computer-based platforms, and virtual reality) [25,26,27]. Interestingly, the use of music therapy and emotional auditory stimulation has been proposed to improve neuroplasticity, motivation, and emotional state [28,29]. Specifically, Leonardi et al. [30] emphasize the potential beneficial effects of music therapy, such as the use of melodic intonation therapy in post-stroke aphasia, suggesting that not only language but also motivation and quality of life can be enhanced. This is consistent with the report of Nadon et al. [31], which has also recently shown that, in healthy subjects, listening to pleasant instrumental music (of both relaxant and stimulating types) is not detrimental for selective attention with respect to a control silence condition, but it may also come to provide protection (i.e., a shielding function) from the distractive quality of noise in the testing setting. Notwithstanding these features, the evidence supporting combined intervention is limited, especially the synergic treatment focused on musical therapy in addition to virtual cognitive training in people with chronic stroke. This gap is critical, as depression, anxiety, and attentional impairments are closely intertwined and best addressed through interventions that are simultaneously emotionally engaging and cognitively demanding [32].

The present study seeks to examine the synergistic effects of Emotional Music Stimulation (EMS) integrated with Virtual Reality Rehabilitation Systems (VRRS) in alleviating non-cognitive symptoms and enhancing functional recovery in post-stroke patients.

## 2. Materials and Methods

### 2.1. Study Setting and Participants

A total of 20 patients with chronic post-stroke conditions (at least six months post-stroke; 10 experimental, 10 control) participated in the study (mean time since onset: 14.2 ± 4.6 months in the experimental group EG and 13.7 ± 5.1 months in the control group CG). The EG (*n* = 10; 4 males/6 females; mean age 51.0 ± 7.29 years and mean education 10.9 ± 2.51) consisted of patients receiving the integrated intervention, while the CG (*n* = 10; 6 males/4 females; mean age 47.6 ± 14.49 years and mean education 9.3 ± 3.20) received only the VRRS intervention. They attended the outpatient clinic of the Neurorehabilitation Unit of IRCCS Neurolesi “Bonino Pulejo” (Messina, Italy) from June 2024 to April 2025.

Stroke patients and/or their caregivers were provided with adequate information about the study and were allowed to participate by providing written consent. The study complied with the principles outlined in the Declaration of Helsinki on Human Rights and was part of a broader multi-year research project registered in Clinicaltrials.gov (ID: NCT07066137), coordinated by the IRCCS Centro Neurolesi “Bonino-Pulejo”. The study was approved by the local Ethics Committee (Protocol code: IRCCSME 47/2023, date: 3 May 2023).

Inclusion criteria were as follows: (1) diagnosis of first ischemic stroke in the chronic phase, i.e., ≥6 months after the event; (2) age range 18–70; (3) absence of disabling sensory impairment (i.e., hearing and visual impairment); and (4) modified Rankin Scale score ≥ 3.

Exclusion criteria were as follows: (1) intake of psychoactive drugs potentially interfering with the training; (2) presence of neurological disorders other than the first ever ischemic stroke; (3) absence of the ability to understand verbal delivery of a simple order, Token Test ≤ 4; and (4) presence of debilitating severe positive psychiatric symptoms such as hallucinations and delusions.

All participants were in the chronic phase of recovery, at least six months post-stroke (mean time since onset: 14.2 ± 4.6 months in the experimental group and 13.7 ± 5.1 months in the control group).

The study specifically targeted post-stroke patients presenting moderate to severe disability (mRS ≥ 3), with predominant cognitive–attentional, motivational, and mood-related deficits. Functional recovery was defined as improvement in global independence and participation, operationalized through reductions in the mRS score.

Lesion characteristics were heterogeneous but predominantly unilateral and ischemic in nature. Eleven participants presented left-hemisphere lesions (55%), and nine had right-hemisphere involvement (45%). The majority of lesions were located within fronto-parietal or temporo-subcortical networks, areas typically associated with attentional and executive functions. Mean NIH Stroke Scale (NIHSS) score at admission was 7.4 ± 2.1, indicating moderate severity. Stroke etiology was ischemic in 85% of patients and hemorrhagic in 15%. This distribution aligns with the clinical profile commonly encountered in chronic post-stroke neurorehabilitation cohorts.

### 2.2. Procedures

Participants in the CG received cognitive training through the VRRS alone, while those in the EG received the same intervention enriched with EMS.

All study participants underwent the same VRRS cognitive rehabilitation, 3 times a week for 8 weeks (i.e., 24 sessions, each lasting about 60 min). The EG was submitted to the EMS in addition to the VRRS system (24 sessions of 45 min each, 3 times a week for 8 weeks). This timeline corresponds to contemporary optimal neurorehabilitation, underlining intense, repeated, and ecologically oriented cognitive stimulation.

Each rehabilitation session followed a standardized three-phase structure: (1) a warm-up phase (5–10 min) focused on orienting attention and re-familiarization with the virtual environment through simple visual–motor tasks; (2) a main training phase (30 min) consisting of individualized VRRS cognitive exercises designed to stimulate selective, sustained, alternating, and divided attention through progressively challenging tasks (e.g., target identification amid distractors, rule-switching, and dual-task paradigms); and (3) a cool-down phase (5–10 min) aimed at consolidation, feedback, and relaxation. During this final phase, the therapist provided performance feedback and emotional support, while patients performed lower-demand activities promoting attention stability and relaxation. The same sessional structure was applied to both groups; in the experimental condition, the beginning and end of each session were accompanied by the patient’s personalized EMS. Each session lasted 45 min of active VRRS cognitive training in both groups; in the experimental group, the total duration was approximately 60 min, including time for music setup, feedback, and individualized adjustment.

### 2.3. Outcome Measures

All stroke patients included in the study were assessed by a multidisciplinary team comprising a neurologist, neuropsychologist, physiotherapist, and speech therapist, both at baseline (T0) and after completion of the rehabilitation protocols (T1). The selection of assessment instruments was guided by the aim of capturing a comprehensive profile of functional, cognitive, emotional, and motivational changes expected to result from the interventions. More specifically, we measured global disability and functioning using the mRS, a scale widely accepted as the standard for measuring the degree of disability and dependence in activities of daily living after neurologic disorders.

Cognitive estimate was determined with the MoCA, a validated instrument that can identify impairment in a wide range of neurocognitive domains, such as attention, memory, language, executive, visuospatial, and visuo-constructional abilities [23]. The Intrinsic Motivation Inventory (IMI) was used to investigate the intrinsic motivation of patients towards the rehabilitation process. This measure is a sophisticated self-report measure of various dimensions of motivation, such as interest, perceived competence, and relatedness [33].

Furthermore, all patients were subjected to non-cognitive examination using the HRS-A and HRS-D by interviewing the patient directly as well as observing their interaction with the clinician to confirm the presence of mood and anxiety symptoms. Finally, to track physiological indices of stress and autonomic control, heart rate (HR) was measured in the experimental and CGs by dedicated nursing staff through the intervention.

A summary description of each outcome measure is detailed in Table 1, including the assessed domain.

### 2.4. Interventions

All subjects of both treatment groups underwent cognitive rehabilitation with the Virtual Reality Rehabilitation System (VRRS; Khymeia Group S.r.l., Padua, Italy), non-immersive computer programs purposefully designed for neurorehabilitation. The VRRS cognitive attention module (software version 3.5) was used to assess and train the key components of attention commonly impacted by ischemic stroke. We used the VRRS cognitive attention module to assess the key components of attention commonly impacted by ischemic stroke (i.e., selective, sustained, alternating, and divided attention). Sessions were delivered in a specialized, clinical setting by trained neurorehabilitation personnel. The VRRS offers a wide range of interactive, game-like digital exercises. Selective attention tasks typically involve rapid identification of target stimuli amid distractors, promoting the ability to filter irrelevant information. Sustained attention was exercised through prolonged vigilance and continuous performance tasks, requiring patients to maintain focus over extended periods. Alternating attention was targeted through activities that necessitated flexible shifts between task rules or stimulus sets, while divided attention was stimulated via dual-task paradigms, demanding simultaneous processing of multiple information streams. Every session was adapted, taking advantage of the VRRS’s capability of modifying on a trial-by-trial basis the level of difficulty of the tasks based on the ongoing performance of the user. Real-time audiovisual feedback enabled enhanced engagement, consolidation of learning, and motivation.

### 2.5. Combined Rehabilitative Approach: VRRS + EMS

For subjects randomized to the EG, EMS was administered concurrently with regular VRRS-based cognitive rehabilitation during each training session. (Table 2).

EMS was delivered through stereo audio systems (speakers). Before the intervention, each patient underwent a semi-structured interview with a certified music therapist to identify familiar and emotionally positive songs frequently listened to during meaningful life moments (e.g., childhood, adolescence, marriage, or family events). When patients were unable to provide this information, interviews were conducted with family members or caregivers. Individualized playlists were then created using these emotionally salient songs without any structural manipulation. The playlists included classical, instrumental, and contemporary pieces. Throughout every VRRS session, EMS rolled without interruption, and the music therapist was observing the patients’ reactions, adjusting volume instantly while keeping the best engagement and the least fatigue. All combined sessions lasted for around 60 min. Task parameters were automatically recorded by the VRRS platform, ensuring objective monitoring of session duration, task completion, accuracy, and response time.

### 2.6. Statistical Analysis

Descriptive statistics are reported for each group and time point (T0 and T1). The Shapiro–Wilk test was used to assess the normality of the variables’ distribution. Non-parametric analysis was performed, and variables were expressed as median and interquartile range (first-third quartile), in accordance with the results of the normality tests and given the limited sample size (*n* = 10 per group). Within-group comparisons (T0 vs. T1) were conducted using Wilcoxon signed-rank tests, while between-group comparisons at each time point (T0 and T1) were performed using Mann–Whitney U tests. Effect sizes (r) were calculated following Rosenthal’s formula (r = Z/√N), as recommended for non-parametric tests (Rosenthal, 1991) [34], and interpreted according to Cohen’s (1988) [35] benchmarks for small (r ≈ 0.1), medium (r ≈ 0.3), and large (r ≥ 0.5) effects. r were calculated for each Wilcoxon or Mann–Whitney U test. In addition to raw scores, delta values (Δ = T1 − T0) were computed for each variable to quantify individual-level change over time. These delta scores were then compared between groups using Wilcoxon tests, and effect sizes were also reported. Furthermore, Spearman correlation analyses were conducted on delta scores to investigate the associations between changes in functional capacity (ΔFC) and changes in affective symptoms (ΔHRS-D and ΔHRS-A). All tests were two-tailed. Statistical analyses were conducted using R (version 4.4.2), with significance set at *p* < 0.05. All statistical analyses were performed at a 95% confidence level. Given the exploratory design and pilot sample size (*n* = 10 per group), no a priori power calculation was performed. Non-parametric tests were chosen to provide robust inference under small-sample conditions and non-normal data distribution.

## 3. Results

### 3.1. Within-Group Comparisons (T0 vs. T1)

In the EG, significant improvements were observed from T0 to T1 in several variables (Table 3). MOCA scores significantly increased (*p* = 0.006, r = 0.87), as did IMI scores (*p* = 0.006, r = 0.87), indicating a large effect. Additionally, significant reductions were found in HRS-D (*p* = 0.005, r = 0.88), HRS-A (*p* = 0.008, r = 0.83), and FC (*p* = 0.002, r = 0.98), all with large effect sizes. The modified Rankin Scale (20) also showed a significant decrease (*p* = 0.004, r = 0.90), confirming clinical improvement in functional status.

In the CG, more modest changes were detected. MOCA scores improved significantly (*p* = 0.007, r = 0.85), indicating a large effect. IMI also increased significantly (*p* < 0.001, r = 0.83), while FC decreased slightly but significantly (*p* = 0.04, r = 0.66). No significant within-group change was observed for HRS-D (*p* = 0.77, r = 0.09), HRS-A (*p* = 0.05, r = 0.63), and the modified Rankin Scale (*p* = 0.15, r = 0.46).

### 3.2. Between-Group Comparisons (T0 vs. T1)

Before analyzing clinical outcomes, baseline demographic variables were compared. No significant differences were observed between the experimental and control groups for age or time since stroke onset (both *p* > 0.05), ensuring comparable demographic and chronicity profiles across groups. At baseline, no statistically significant differences were observed between the experimental and CGs in most variables (Table 3). Specifically, MOCA scores did not differ significantly (*p* = 0.60; r = 0.12), nor did HRS-D (*p* = 0.49; r = 0.15), HRS-A (*p* = 0.76; r = 0.07), IMI (*p* = 0.88; r = 0.03), and modified Rankin Scale scores (*p* = 0.73; r = 0.08), indicating good baseline comparability between groups on cognitive, emotional, and functional measures. The only variable showing a significant between-groups difference at T0 was FC, with the EG showing higher baseline values compared to the CG (*p* = 0.01, r = 0.57), reflecting a large effect size.

At follow-up (T1), significant differences emerged between the experimental and CG in multiple outcome measures (Table 4). MOCA scores were significantly higher in the EG compared to the CG (*p* = 0.005), with a large effect size (r = 0.62), indicating a substantial cognitive improvement attributable to the intervention. Similarly, IMI scores were significantly greater in the EG (*p* < 0.001, r = 0.84), also reflecting a substantial effect on intrinsic motivation. A significant difference was also found in the modified Rankin Scale (*p* = 0.02, r = 0.51), suggesting better functional recovery in the EG. Although HRS-D showed a small effect size (r = 0.27), the group difference did not reach statistical significance (*p* = 0.49). HRS-A differences were not statistically significant (*p* = 0.17), despite a medium effect size (r = 0.31), likely due to sample size constraints. No difference was observed in FC (*p* = 0.85, r = 0.04).

### 3.3. Between-Group Comparisons of Change Scores (ΔT1–T0)

Change scores revealed significantly greater improvements in the EG compared to the CG across all outcome measures (Table 4). Specifically, MoCA scores increased significantly more in the EG than in the CG (*p* = 0.004, r = 0.64), indicating a large effect size. Significant group differences were also observed for affective measures. The EG showed larger reductions in HRS-D (*p* < 0.001, r = 0.83) and HRS-A (*p* = 0.003, r = 0.66), both associated with large effect sizes. In the EG, FC showed a greater reduction compared to the CG (*p* < 0.001, r = 0.98), again reflecting a large effect. A similar pattern was observed for IMI, where the EG exhibited a significantly greater improvement in memory scores (*p* < 0.001, r = 0.84), indicating an exceptionally large effect size. Finally, the modified Rankin Scale (20) also showed significantly greater improvement in the EG compared to the CG (*p* < 0.001, r = 0.74), highlighting a large effect size.

### 3.4. Correlations Between Autonomic Change and Non-Cognitive Symptoms

**Figure 1:**
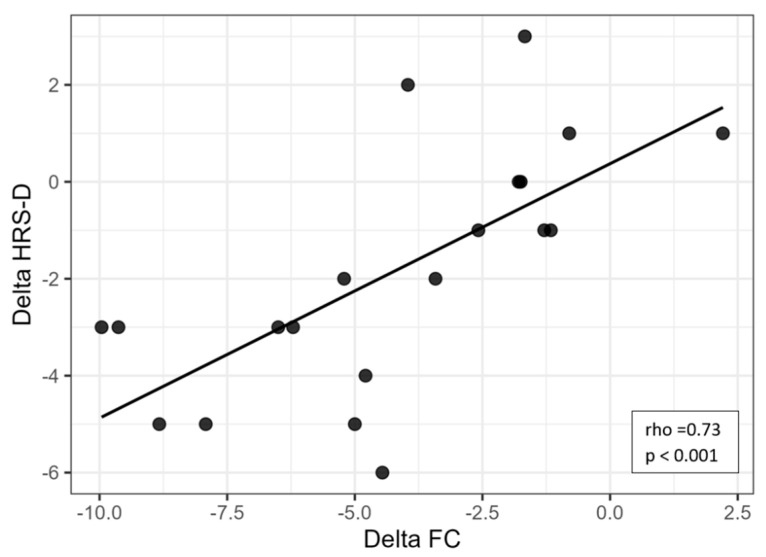
Scatter plot showing the relationship between changes in heart rate (ΔFC) and changes in depressive symptoms (ΔHRS-D). Legend: HRS-D = Hamilton Rating Scale for Depression; FC = frequency of cardiac activity.

**Figure 2:**
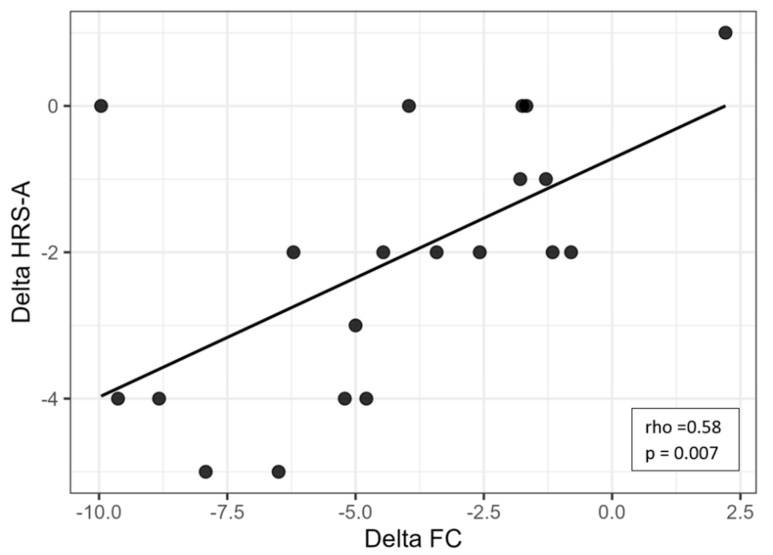
Scatter plot showing the relationship between changes in heart rate (ΔFC) and changes in depressive symptoms (ΔHRS-A). Legend: HRS-A = Hamilton Rating Scale for Anxiety; FC = frequency of cardiac activity.

Correlation analyses were conducted to explore the associations between changes in functional capacity and affective symptoms. A strong and significant positive correlation was found between ΔFC and ΔHRS-D (rho = 0.73, *p* < 0.001), indicating that greater reductions in depressive symptoms were associated with greater improvements in functional capacity (Figure 1). Similarly, a moderate-to-strong positive correlation was observed between ΔFC and ΔHRS-A (rho = 0.58, *p* = 0.007), suggesting a meaningful association between decreased anxiety and improved functionality (Figure 2).

## 4. Discussion

To our knowledge, this quasi-randomized trial is the first study to evaluate a therapist-personalized, concurrent EMS intervention integrated with a standardized VRRS attention training program in individuals with chronic post-stroke conditions. The study assessed multiple outcomes: cognition, motivation, mood, anxiety, functional status, and heart rate as an autonomic marker. Previous research has typically examined either music-based or VR-based cognitive training alone, focused primarily on subacute stroke populations, and seldom incorporated motivational outcomes or autonomic indices alongside clinical assessments [29,36]. By embedding a session-long EMS protocol into each VRRS session, this study addressed a critical question: whether emotionally engaging sensory input can potentiate attention-based rehabilitation beyond the spontaneous recovery window. Both groups improved cognitively, consistent with established evidence on VR-based attention training, but the EMS group showed greater and more consistent gains. This suggests that the difference was not due to therapist interaction or training intensity, which were identical, but to the emotional engagement provided by personalized music. Acting as an “attentional scaffold,” EMS likely stabilized arousal and enhanced task focus, promoting deeper cognitive processing. These results are in line with meta-analytic findings showing VR efficacy in post-stroke cognition [36] and extend previous work in TBI rehabilitation [37], demonstrating an additive benefit when EMS accompanies cognitive training. Motivational data further support this interpretation. Although intrinsic motivation improved in both groups, the increase was markedly higher in the EMS condition. The enhancement of interest, enjoyment, and perceived competence—core IMI domains—provides a plausible mechanism through which EMS may foster cognitive transfer and engagement. This aligns with broader evidence that emotionally pleasant music enhances sustained attention and buffers distraction [38,39]. Symptoms of depression and anxiety also declined significantly in the EMS group, with large effect sizes in change scores despite modest between-group differences at post-test, likely due to limited power. Clinically, these affective improvements are meaningful: reduced emotional distress enhances participation and persistence in therapy. From a neurophysiological perspective, EMS may support emotion regulation by engaging limbic–prefrontal pathways involved in stress modulation, consistent with previous post-stroke music-listening studies showing improved mood and cognitive recovery [40,41,42]. Physiological findings parallel these effects. Heart rate (HR) and frequency of cardiac activity (FC) decreased in both groups, but more sharply in the EMS group. Reductions in FC correlated strongly with improvements in depression and anxiety, suggesting enhanced autonomic regulation during training. Although FC is a coarse index, this pattern indicates a general improvement in physiological balance rather than specific vagal modulation. These results align with research showing that music modulates autonomic activity and promotes psychophysiological stability [43,44,45,46,47].

Functional status mirrored these upstream effects. Only the EMS group improved significantly on the mRS, and even a one-point gain represents meaningful independence in chronic stroke. Although mediation analyses were not feasible, the coherent pattern across motivation, attention, affect, and physiology suggests a plausible pathway to functional benefit. Both groups received identical VRRS dosing by the same multidisciplinary team, isolating EMS as the differentiating factor. Nevertheless, expectancy effects cannot be entirely excluded. Personalized music is inherently engaging and may enhance perceived support. Future studies should include emotionally neutral or mismatched music as auditory controls to disentangle specific from non-specific effects. Our findings align with previous evidence indicating that VR-based rehabilitation can enhance activity and participation following stroke [36], and they complement prior clinical observations suggesting that music can support rehabilitation engagement and daily functioning after brain injury [48,49]. This study’s strengths include an active comparator, standardized dosing, structured EMS delivery, and convergence across clinical and physiological outcomes. Groups were comparable for age and chronicity, minimizing confounding. Limitations include quasi-randomization, small single-center sample size, lack of blinding, and absence of an active auditory control. The small sample limits power and increases Type II error risk, so results should be considered exploratory. Moreover, autonomic assessment was based on HR rather than HRV, and music parameters were tailored individually rather than experimentally controlled. Although the duration of active cognitive training was identical across groups, total session time was slightly longer in the EMS condition due to setup and feedback phases, which should be considered when interpreting the observed effects. Despite these limitations, the consistent pattern of large improvements across domains supports the feasibility and potential of EMS as an adjunct to VR-based cognitive rehabilitation. Larger randomized controlled trials with concealed allocation, blinded assessment, and long-term follow-up are warranted to confirm these preliminary findings.

## 5. Conclusions

Our findings underscore that integrating personalized EMS with a standardized VRRS attention program enhanced cognitive performance, intrinsic motivation, affective balance, autonomic regulation, and overall functional status in chronic stroke patients compared with VRRS alone.

This study demonstrates that the combination of emotional music stimulation and virtual-reality attention training produces a synergistic enhancement across cognitive, motivational, emotional, and autonomic domains. These findings highlight the potential of emotionally enriched digital rehabilitation to extend the benefits of therapy beyond the conventional recovery window.

## Figures and Tables

**Table 1:** Description of outcome measures and assessed domains.

Test/Scale	Specific Domains Assessed	Description
Modified Rankin Scale (mRS) [20]	Global disability, Activities of daily living, and social participation.	The mRS is a clinician-administered scale widely used to assess the degree of disability or dependence in daily activities among people who have suffered a stroke or other neurological events. Scores range from 0 (no symptoms) to 6 (death), with higher scores reflecting greater disability. It provides a robust index of overall functional outcome and social integration.
Montreal Cognitive Assessment (MoCA) [23]	Visuospatial/executive function; Naming; Memory; Attention; Language; Abstraction; Delayed recall; Orientation	The MoCA is a rapid screening tool for mild cognitive impairment, assessing several neuropsychological domains, including visuospatial abilities, executive functioning, short-term memory recall, attention, concentration, working memory, language, abstraction, and orientation to time and place. The total score ranges from 0 to 30; higher scores indicate better cognition.
Intrinsic Motivation Inventory (IMI) [33]	Interest/enjoyment; Perceived competence; Perceived choice; Effort/importance; Pressure/tension; Value/usefulness; relatedness.	The IMI is a multidimensional self-report instrument that assesses the individual’s subjective experience related to a target activity, specifically intrinsic motivation. It covers domains such as personal interest and enjoyment, perceived competence in the activity, exerted effort, perceived value and usefulness, felt pressure or tension, and sense of relatedness to others.
Hamilton Rating Scale for Anxiety (HRS-A) [21]	Psychic anxiety (mental agitation, psychological distress); Somatic anxiety (physical complaints related to anxiety)	The HRS-A is a clinician-rated scale for quantifying the severity of a patient’s anxiety. It comprises 14 items, each rated on a 5-point scale, evaluating both psychic (subjective, mental) and somatic (physical, autonomic) symptoms of anxiety. Higher total scores indicate more severe anxiety. The scale is administered via clinical observation and interview.
Hamilton Rating Scale for Depression (HRS-D) [22]	Depressed mood; Feelings of guilt; Suicidal ideation; Insomnia; Work and activities; Retardation/agitation; Anxiety; Somatic symptoms	The HRS-D is a clinician-administered scale designed to assess the presence and severity of depressive symptoms across multiple domains, including affective, cognitive, behavioral, and somatic aspects. The 21-item scale covers core symptoms such as mood, guilt, sleep, activity, psychomotor changes, anxiety, and somatic complaints. Higher scores indicate more severe depression.
Frequency of Cardiac Activity (FC)	Heart rate; Autonomic function; Physiological stress response.	FC is monitored to provide an index of autonomic nervous system activity, specifically heart rate, which reflects physiological arousal and stress response. Heart rate was measured in both groups by nursing and neurophysiology staff to monitor for potential autonomic changes associated with intervention or rehabilitation sessions.

**Table 2:** Core components of the EMS program.

Component	Description
Personalized Music Selection	Patients listen to music that is emotionally meaningful or familiar to them (e.g., from their youth, about significant events such as marriage, graduation, childbirth…). This evokes stronger emotional and memory-related brain activity.
Guided Listening Sessions	Structured sessions led by therapists where patients reflect on their emotions during or after listening.
Live or Interactive Music	Use of live instruments, singing, or rhythmic participation (e.g., drumming) to enhance emotional engagement.
Music-Based Relaxation	Slow, calming music used for relaxation, stress reduction, and autonomic regulation.
Emotion Identification Tasks	Patients listen to music of different emotional tones (e.g., happy, sad, tense) and identify the feelings, promoting motivation and emotional awareness.

**Table 3:** Within- and between-group analysis for all outcome measures at baseline (T0) and post-intervention (T1).

		Experimental	Control		
		Median (I–III Quartile)	Median (I–III Quartile)	*p*	Effect Size
MOCA	T0	12.0 (11.25–12.0)	12.0 (12.0–12.75)	0.60	r = 0.12
	T1	17.0 (15.25–17.0)	14.0 (13.0–14.0)	0.005 *	r = 0.62
	*p*	0.006 *	0.007 *		
HRS-D	T0	12.5 (11.0–17.50)	12.0 (10.25–14.5)	0.49	r = 0.15
	T1	9.5 (7.25–13.75	12.5 (10.5–13.0)	0.22	r = 0.27
	*p*	0.005 *	0.77		
HRS-A	T0	19.5 (18.25–22.75)	19.5 (18.0–21.75)	0.76	r = 0.07
	T1	16.0 (16.0–18.5)	19.0 (17.25–21.5)	0.17	r = 0.31
	*p*	0.008 *	0.05		
FC	T0	81.5 (79.18–84.43)	75.12 (73.27–78.15)	0.01 *	r = 0.57
	T1	74.04 (73.99–75.79)	74.18 (72.42–76.58)	0.85	r = 0.04
	*p*	0.002 *	0.04 *		
IMI	T0	165.0 (151.25–172.50)	160.0 (156.25–173.75)	0.88	r = 0.03
	T1	210.0 (205.0–225.0)	172.5 (165.0–180.0)	<0.001 *	r = 0.84
	*p*	0.006 *	0.008 *		
Modified Rankin Scale	T0	4.0 (4.0–5.0)	4.0 (4.0–4.75)	0.73	r = 0.08
	T1	3.0 (2.25–3.0)	4.0 (3.0–4.75)	0.02 *	r = 0.51
	*p*	0.004 *	0.15		

* *p* < 0.05. Effect sizes are expressed as r for non-parametric tests. Legend: MOCA = Montreal Cognitive Assessment; HRS-D = Hamilton Rating Scale for Depression; HRS-A = Hamilton Rating Scale for Anxiety; FC = frequency of cardiac activity; IMI = Intrinsic Motivation Inventory.

**Table 4:** Between-group comparisons of change scores in clinical, cognitive, emotional, and physiological measures.

	Experimental	Control		
	Median (I–III Quartile)	Median (I–III Quartile)	*p*-Value	Effect Size
ΔMOCA	4.5 (3.25, 5.0)	2.0 (1.25, 2.0)	0.004 *	r = 0.64
ΔHRS-D	−3.5 (−5.0, −3.0)	0.0 (−1.0, 1.0)	<0.001 *	r = 0.83
ΔHRS-A	−4.0 (−4.0, −2.25)	−1.0 (−2.0, 0.0)	0.003 *	r = 0.66
ΔFC	−6.35 (−8.60, −5.05)	−1.71 (−2.38, −1.19)	<0.001 *	r = 0.98
ΔIMI	54.0 (41.25, 60.0)	10.0 (5.0, 10.0)	<0.001 *	r = 0.84
ΔmRS	−1.0 (−2.0, −1.0)	0.0 (−0.75, 0.0)	<0.001 *	r = 0.74

* *p* < 0.05. Effect sizes are expressed as r for non-parametric tests. Legend: MOCA = Montreal Cognitive Assessment; HRS-D = Hamilton Rating Scale for Depression; HRS-A = Hamilton Rating Scale for Anxiety; FC = frequency of cardiac activity; IMI = Intrinsic Motivation Inventory.

## Data Availability

The data presented in this study are available on request from the corresponding author due to privacy and ethical reasons.
